# Poly[tetra­aqua­(μ_4_-benzene-1,3,5-tri­carboxyl­ato)sodium(I)zinc(II)]

**DOI:** 10.1107/S1600536810009232

**Published:** 2010-03-17

**Authors:** Chao-Hong Ma, Xiu-Yan Wang, Xian-Wu Dong, Yu-Jie Li

**Affiliations:** a Jilin Agricultural Science and Technology College, Jilin 132101, People’s Republic of China

## Abstract

In the title compound, [NaZn(C_9_H_3_O_6_)(H_2_O)_4_]_*n*_, the Zn^II^ atom is six-coordinated by four O atoms from two different benzene-1,3,5-tricarboxyl­ate anions and two water O atoms in a distorted tetragonal-bipyramidal geometry and the Na^I^ atom is five-coordinated by three O atoms from three different benzene-1,3,5-tricarboxyl­ate anions and two water O atoms in a distorted trigonal-bipyramidal geometry. The benzene-1,3,5-tricarboxyl­ate anion bridges two Zn^II^ atoms and two Na^I^ atoms, resulting in the formation of a two-dimensional layer structure. Inter­molecular O—H⋯O hydrogen-bonding inter­actions generate a three-dimensional superamolecular structure.

## Related literature

For related sructures, see: Chui *et al.* (1999 ▶); Majumder *et al.* (2005 ▶).
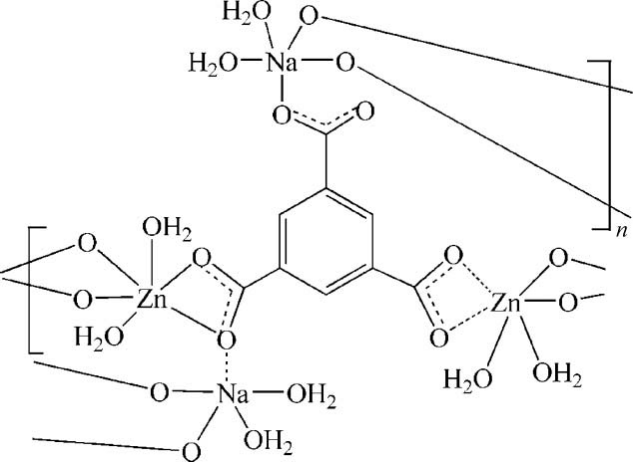


## Experimental

### 

#### Crystal data

[NaZn(C_9_H_3_O_6_)(H_2_O)_4_]
*M* *_r_* = 367.54
Monoclinic, 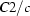

*a* = 23.425 (5) Å
*b* = 10.146 (2) Å
*c* = 14.427 (3) Å
β = 126.50 (3)°
*V* = 2756.3 (15) Å^3^
*Z* = 8
Mo *K*α radiation
μ = 1.86 mm^−1^
*T* = 293 K
0.23 × 0.22 × 0.20 mm

#### Data collection

Oxford Diffraction Gemini R Ultra diffractometer
Absorption correction: multi-scan (*CrysAlis PRO*; Oxford Diffraction, 2007 ▶) *T*_min_ = 0.765, *T*_max_ = 0.876
13077 measured reflections
3348 independent reflections
2119 reflections with *I* > 2σ(*I*)
*R*_int_ = 0.056

#### Refinement

*R*[*F* ^2^ > 2σ(*F* ^2^)] = 0.065
*wR*(*F* ^2^) = 0.187
*S* = 0.99
3348 reflections
214 parameters
106 restraints
H atoms treated by a mixture of independent and constrained refinement
Δρ_max_ = 1.68 e Å^−3^
Δρ_min_ = −0.79 e Å^−3^

### 

Data collection: *CrysAlis PRO* (Oxford Diffraction, 2007 ▶); cell refinement: *CrysAlis RED* (Oxford Diffraction, 2007 ▶); data reduction: *CrysAlis RED*; program(s) used to solve structure: *SHELXS97* (Sheldrick, 2008 ▶); program(s) used to refine structure: *SHELXL97* (Sheldrick, 2008 ▶); molecular graphics: *SHELXTL* (Sheldrick, 2008 ▶); software used to prepare material for publication: *SHELXTL* and *DIAMOND* (Brandenburg, 1998 ▶).

## Supplementary Material

Crystal structure: contains datablocks global, I. DOI: 10.1107/S1600536810009232/jj2022sup1.cif

Structure factors: contains datablocks I. DOI: 10.1107/S1600536810009232/jj2022Isup2.hkl

Additional supplementary materials:  crystallographic information; 3D view; checkCIF report

## Figures and Tables

**Table 1:** Hydrogen-bond geometry (Å, °)

*D*—H⋯*A*	*D*—H	H⋯*A*	*D*⋯*A*	*D*—H⋯*A*
O1*W*—H1*WA*⋯O5^i^	0.93 (5)	2.35 (8)	3.090 (6)	137 (9)
O2*W*—H2*WA*⋯O2^ii^	0.93 (5)	2.28 (9)	2.998 (6)	133 (9)
O3*W*—H3*WA*⋯O6	0.85 (5)	2.11 (7)	2.747 (7)	132 (7)
O4*W*—H4*WB*⋯O4^iii^	1.00 (5)	1.71 (5)	2.686 (8)	166 (8)
O4*W*—H4*WA*⋯O3*W*^iv^	0.85 (4)	2.37 (7)	2.948 (8)	126 (9)

Symmetry codes: (i) 
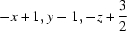
; (ii) 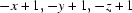; (iii) 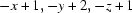; (iv) 
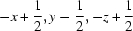
.

**Fig. 1.**
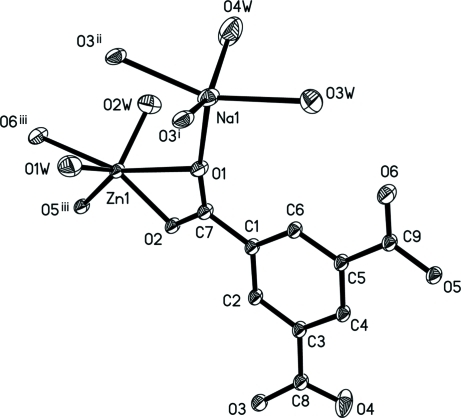
ORTEP diagram of the coordination environments for ZnII atom and NaI atom in (I), showing 30% probability displacement ellipsoids, crystalline water molecules and the atomic numbering scheme [symmetry code: (i) 1-x, y, 1.5-z; (ii) x-0.5, 1.5-y, z-0.5; (iii) x, y-1, z]. H atoms have been omitted for clarity.

**Fig. 2.**
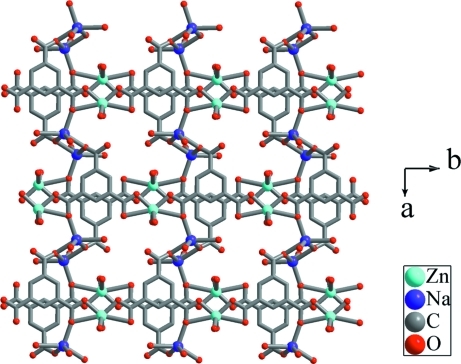
The two dimensional layer of (I). The H atoms have been omitted.

**Fig. 3.**
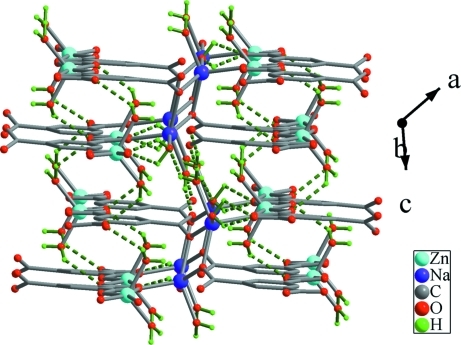
The supramolecular framework of I.

## supplementary crystallographic information

### Comment

In the title compound, (I), each Zn^II^ cation is six-coordinated by five O atoms from two different benzene-1,3,5-tricarboxylate anions and two water molecules. The Zn—O (carboxylate) distance in I is similar to the equivalent value in a related compounds (Majumder *et al.* 2005). Each Na^I^ cation is five coordinated by three oxygen atoms from three different benzene-1,3,5-tricarboxylate anions and two water molecules. The Na—O (carboxylate) distance is similar to the related compounds (Chui *et al.* 1999) (Fig. 1). The Zn^II^ centers and the Na^I^ centers are bridged by benzene-1,3,5-tricarboxylate anions, resulting in a two dimensional layer (Fig. 2). In (I), there are intra and intermolecular O-H···O hydrogen bonds involving the water molecules and the oxygen atoms of the carboxylate groups (Table 1). The adjacent layers are bridged by the hydrogen bonds, and the whole structure displays a three dimensional supramolecular framework (Fig. 3).

### Experimental

The mixture of of benzene-1,3,5-tricarboxylate acid (0.063 g, 0.3 mmol), NaOH (0.024 g, 0.25 mmol), Zn(Ac)_2_ (0.066 g, 0.3 mmol), and 10 ml H_2_O was sealed in 18 ml Teflon-lined stainless steel container. The container was heated to 150 °C and held at that temperature for 72 h, then cooled to room temperature at a rate of 10 °C.h^-1^. And then crystals of the title compound were isolated.

### Refinement

C-bound H-atoms were geometrically positioned (C—H = 0.93 Å) and refined using a riding model, with U_iso_(H) = 1.2U_eq_ (C). The H atoms of the water molecules were located in a difference map, and were refined with distance restraints of O—H = 0.85 Å.

### Crystal data

**Table:**

[NaZn(C_9_H_3_O_6_)(H_2_O)_4_]	*F*(000) = 1488
*M**_r_* = 367.54	*D*_x_ = 1.771 Mg m^−^^3^
Monoclinic, *C*2/*c*	Mo *K*α radiation, λ = 0.71073 Å
Hall symbol: -C 2yc	Cell parameters from 3348 reflections
*a* = 23.425 (5) Å	θ = 4.7–29.2°
*b* = 10.146 (2) Å	µ = 1.86 mm^−^^1^
*c* = 14.427 (3) Å	*T* = 293 K
β = 126.50 (3)°	Block, colorless
*V* = 2756.3 (15) Å^3^	0.23 × 0.22 × 0.20 mm
*Z* = 8	

### Data collection

**Table:**

Oxford Diffraction Gemini R Ultra diffractometer	3348 independent reflections
Radiation source: fine-focus sealed tube	2119 reflections with *I* > 2σ(*I*)
graphite	*R*_int_ = 0.056
Detector resolution: 10.0 pixels mm^-1^	θ_max_ = 29.2°, θ_min_ = 4.7°
ω scan	*h* = −31→29
Absorption correction: multi-scan (*CrysAlis PRO*; Oxford Diffraction, 2007)	*k* = −13→13
*T*_min_ = 0.765, *T*_max_ = 0.876	*l* = −19→15
13077 measured reflections	

### Refinement

**Table:**

Refinement on *F*^2^	Primary atom site location: structure-invariant direct methods
Least-squares matrix: full	Secondary atom site location: difference Fourier map
*R*[*F*^2^ > 2σ(*F*^2^)] = 0.065	Hydrogen site location: inferred from neighbouring sites
*wR*(*F*^2^) = 0.187	H atoms treated by a mixture of independent and constrained refinement
*S* = 0.99	*w* = 1/[σ^2^(*F*_o_^2^) + (0.1186*P*)^2^] where *P* = (*F*_o_^2^ + 2*F*_c_^2^)/3
3348 reflections	(Δ/σ)_max_ < 0.001
214 parameters	Δρ_max_ = 1.68 e Å^−^^3^
106 restraints	Δρ_min_ = −0.79 e Å^−^^3^

### Special details

**Table:**

Geometry. All esds (except the esd in the dihedral angle between two l.s. planes) are estimated using the full covariance matrix. The cell esds are taken into account individually in the estimation of esds in distances, angles and torsion angles; correlations between esds in cell parameters are only used when they are defined by crystal symmetry. An approximate (isotropic) treatment of cell esds is used for estimating esds involving l.s. planes.
Refinement. Refinement of *F*^2^ against ALL reflections. The weighted *R*-factor wR and goodness of fit *S* are based on *F*^2^, conventional *R*-factors *R* are based on *F*, with *F* set to zero for negative *F*^2^. The threshold expression of *F*^2^ > σ(*F*^2^) is used only for calculating *R*-factors(gt) etc. and is not relevant to the choice of reflections for refinement. *R*-factors based on *F*^2^ are statistically about twice as large as those based on *F*, and *R*- factors based on ALL data will be even larger.

### Fractional atomic coordinates and isotropic or equivalent isotropic displacement parameters (Å^2^)

**Table:**

	*x*	*y*	*z*	*U*_iso_*/*U*_eq_	
Zn1	0.44260 (3)	0.47289 (5)	0.56752 (5)	0.0277 (2)	
Na1	0.29885 (10)	0.8061 (2)	0.4465 (2)	0.0406 (5)	
C9	0.4802 (2)	1.2199 (5)	0.5988 (4)	0.0284 (7)	
C8	0.7090 (3)	0.9737 (5)	0.8706 (5)	0.0331 (7)	
C4	0.5942 (2)	1.0936 (5)	0.7306 (4)	0.0268 (6)	
H008	0.6191	1.1727	0.7565	0.032*	
C6	0.4854 (3)	0.9745 (4)	0.6091 (4)	0.0264 (6)	
H012	0.4365	0.9746	0.5531	0.032*	
C5	0.5209 (2)	1.0942 (4)	0.6463 (4)	0.0265 (6)	
C3	0.6303 (3)	0.9744 (5)	0.7762 (4)	0.0280 (6)	
C2	0.5932 (2)	0.8565 (5)	0.7375 (4)	0.0267 (7)	
H017	0.6173	0.7773	0.7687	0.032*	
C7	0.4788 (2)	0.7306 (5)	0.6098 (4)	0.0289 (6)	
C1	0.5198 (2)	0.8558 (5)	0.6519 (4)	0.0263 (6)	
O1	0.41242 (17)	0.7350 (3)	0.5455 (3)	0.0344 (7)	
O1W	0.3853 (3)	0.4688 (5)	0.6293 (5)	0.0575 (12)	
O2W	0.3838 (2)	0.4876 (6)	0.3961 (4)	0.0546 (12)	
O4W	0.2642 (4)	0.7532 (7)	0.2655 (5)	0.094 (2)	
O6	0.41411 (19)	1.2178 (4)	0.5267 (4)	0.0448 (10)	
O3W	0.3018 (3)	1.0451 (6)	0.4118 (6)	0.087 (2)	
O5	0.51477 (17)	1.3279 (3)	0.6362 (3)	0.0316 (7)	
O2	0.51282 (16)	0.6223 (3)	0.6419 (3)	0.0292 (6)	
O3	0.73536 (18)	0.8792 (4)	0.9374 (3)	0.0376 (7)	
O4	0.7451 (2)	1.0708 (6)	0.8821 (6)	0.092 (2)	
H1WA	0.392 (5)	0.402 (9)	0.679 (8)	0.138*	
H4WA	0.224 (4)	0.730 (11)	0.207 (3)	0.138*	
H4WB	0.269 (5)	0.822 (8)	0.221 (4)	0.138*	
H3WA	0.331 (5)	1.086 (2)	0.475 (5)	0.138*	
H3WB	0.336 (5)	1.053 (3)	0.394 (10)	0.138*	
H2WB	0.363 (6)	0.559 (8)	0.345 (8)	0.138*	
H1WB	0.340 (3)	0.471 (11)	0.579 (8)	0.138*	
H2WA	0.389 (6)	0.440 (10)	0.347 (8)	0.138*	

### Atomic displacement parameters (Å^2^)

**Table:**

	*U*^11^	*U*^22^	*U*^33^	*U*^12^	*U*^13^	*U*^23^
Zn1	0.0207 (3)	0.0165 (3)	0.0349 (4)	0.0010 (2)	0.0105 (3)	0.0011 (2)
Na1	0.0259 (10)	0.0345 (12)	0.0480 (13)	0.0002 (9)	0.0148 (10)	−0.0034 (10)
C9	0.0229 (10)	0.0168 (11)	0.0363 (11)	0.0020 (9)	0.0126 (9)	0.0020 (9)
C8	0.0241 (11)	0.0219 (11)	0.0384 (12)	0.0024 (10)	0.0104 (10)	0.0052 (10)
C4	0.0220 (9)	0.0167 (10)	0.0352 (10)	0.0018 (9)	0.0134 (9)	0.0029 (9)
C6	0.0213 (9)	0.0165 (10)	0.0352 (10)	0.0021 (8)	0.0135 (8)	0.0016 (9)
C5	0.0219 (9)	0.0162 (10)	0.0350 (10)	0.0020 (8)	0.0135 (8)	0.0023 (8)
C3	0.0220 (9)	0.0182 (10)	0.0357 (10)	0.0022 (8)	0.0129 (8)	0.0037 (8)
C2	0.0214 (9)	0.0168 (10)	0.0355 (10)	0.0025 (8)	0.0134 (9)	0.0028 (9)
C7	0.0215 (9)	0.0174 (9)	0.0382 (10)	0.0019 (8)	0.0126 (8)	0.0003 (9)
C1	0.0211 (9)	0.0164 (9)	0.0356 (10)	0.0022 (8)	0.0138 (8)	0.0015 (8)
O1	0.0224 (11)	0.0196 (11)	0.0428 (13)	0.0022 (9)	0.0094 (10)	−0.0011 (10)
O1W	0.040 (2)	0.069 (3)	0.054 (3)	0.002 (2)	0.023 (2)	0.007 (2)
O2W	0.045 (2)	0.063 (3)	0.042 (3)	0.005 (2)	0.018 (2)	0.000 (2)
O4W	0.127 (5)	0.088 (4)	0.063 (4)	−0.059 (4)	0.055 (4)	−0.023 (3)
O6	0.0245 (18)	0.0215 (19)	0.060 (3)	0.0045 (15)	0.0098 (18)	0.0032 (18)
O3W	0.054 (3)	0.060 (4)	0.085 (4)	−0.004 (2)	0.007 (3)	0.032 (3)
O5	0.0242 (11)	0.0171 (12)	0.0383 (13)	0.0013 (10)	0.0104 (10)	0.0017 (10)
O2	0.0219 (10)	0.0163 (11)	0.0388 (12)	0.0010 (9)	0.0123 (9)	0.0004 (10)
O3	0.0263 (12)	0.0251 (12)	0.0402 (13)	0.0029 (10)	0.0084 (10)	0.0066 (11)
O4	0.029 (2)	0.058 (3)	0.119 (5)	−0.010 (2)	0.006 (3)	0.044 (3)

### Geometric parameters (Å, °)

**Table:**

Zn1—O2W	1.996 (5)	C6—C5	1.387 (7)
Zn1—O5^i^	2.002 (3)	C6—H012	0.9300
Zn1—O1W	2.006 (5)	C3—C2	1.385 (7)
Zn1—O2	2.014 (3)	C2—C1	1.400 (6)
Na1—O1	2.262 (4)	C2—H017	0.9300
Na1—O4W	2.290 (6)	C7—O1	1.251 (6)
Na1—O3^ii^	2.352 (4)	C7—O2	1.271 (6)
Na1—O3^iii^	2.365 (5)	C7—C1	1.486 (7)
Na1—O3W	2.486 (5)	O1W—H1WA	0.93 (5)
Na1—Na1^iv^	3.621 (4)	O1W—H1WB	0.86 (5)
C9—O6	1.250 (6)	O2W—H2WB	0.94 (5)
C9—O5	1.277 (6)	O2W—H2WA	0.93 (5)
C9—C5	1.493 (6)	O4W—H4WA	0.85 (4)
C8—O3	1.233 (6)	O4W—H4WB	1.00 (5)
C8—O4	1.245 (7)	O3W—H3WA	0.85 (5)
C8—C3	1.505 (7)	O3W—H3WB	0.98 (5)
C4—C5	1.394 (7)	O5—Zn1^v^	2.002 (3)
C4—C3	1.396 (7)	O3—Na1^vi^	2.352 (4)
C4—H008	0.9300	O3—Na1^iii^	2.365 (5)
C6—C1	1.374 (6)		
			
O2W—Zn1—O5^i^	115.16 (19)	C6—C5—C4	118.5 (4)
O2W—Zn1—O1W	113.7 (2)	C6—C5—C9	119.9 (4)
O5^i^—Zn1—O1W	110.88 (18)	C4—C5—C9	121.6 (4)
O2W—Zn1—O2	110.30 (19)	C2—C3—C4	119.9 (4)
O5^i^—Zn1—O2	96.19 (14)	C2—C3—C8	119.8 (4)
O1W—Zn1—O2	109.20 (18)	C4—C3—C8	120.2 (4)
O1—Na1—O4W	97.5 (2)	C3—C2—C1	120.5 (4)
O1—Na1—O3^ii^	104.33 (15)	C3—C2—H017	119.8
O4W—Na1—O3^ii^	88.02 (18)	C1—C2—H017	119.8
O1—Na1—O3^iii^	114.83 (16)	O1—C7—O2	122.3 (4)
O4W—Na1—O3^iii^	147.3 (2)	O1—C7—C1	119.2 (4)
O3^ii^—Na1—O3^iii^	79.70 (15)	O2—C7—C1	118.5 (4)
O1—Na1—O3W	105.94 (18)	C6—C1—C2	118.4 (4)
O4W—Na1—O3W	91.8 (3)	C6—C1—C7	120.2 (4)
O3^ii^—Na1—O3W	149.48 (19)	C2—C1—C7	121.4 (4)
O3^iii^—Na1—O3W	84.2 (2)	C7—O1—Na1	162.9 (3)
O1—Na1—Na1^iv^	115.79 (15)	Zn1—O1W—H1WA	120 (7)
O4W—Na1—Na1^iv^	121.80 (18)	Zn1—O1W—H1WB	116 (8)
O3^ii^—Na1—Na1^iv^	39.98 (10)	H1WA—O1W—H1WB	104 (7)
O3^iii^—Na1—Na1^iv^	39.72 (10)	Zn1—O2W—H2WB	134 (7)
O3W—Na1—Na1^iv^	119.6 (2)	Zn1—O2W—H2WA	127 (7)
O6—C9—O5	121.8 (4)	H2WB—O2W—H2WA	92 (6)
O6—C9—C5	120.3 (4)	Na1—O4W—H4WA	129 (4)
O5—C9—C5	117.9 (4)	Na1—O4W—H4WB	118 (3)
O3—C8—O4	121.8 (5)	H4WA—O4W—H4WB	92 (5)
O3—C8—C3	119.1 (4)	Na1—O3W—H3WA	111 (3)
O4—C8—C3	119.1 (5)	Na1—O3W—H3WB	105 (2)
C5—C4—C3	120.1 (4)	H3WA—O3W—H3WB	89 (6)
C5—C4—H008	119.9	C9—O5—Zn1^v^	106.5 (3)
C3—C4—H008	119.9	C7—O2—Zn1	108.7 (3)
C1—C6—C5	122.5 (4)	C8—O3—Na1^vi^	132.2 (4)
C1—C6—H012	118.7	C8—O3—Na1^iii^	125.0 (4)
C5—C6—H012	118.7	Na1^vi^—O3—Na1^iii^	100.30 (15)
			
C1—C6—C5—C4	−0.2 (8)	O2—C7—C1—C6	172.8 (5)
C1—C6—C5—C9	−178.2 (5)	O1—C7—C1—C2	170.0 (5)
C3—C4—C5—C6	−0.3 (7)	O2—C7—C1—C2	−9.0 (8)
C3—C4—C5—C9	177.6 (5)	O2—C7—O1—Na1	166.7 (9)
O6—C9—C5—C6	0.8 (8)	C1—C7—O1—Na1	−12.3 (16)
O5—C9—C5—C6	179.3 (5)	O4W—Na1—O1—C7	121.7 (13)
O6—C9—C5—C4	−177.1 (5)	O3^ii^—Na1—O1—C7	−148.4 (13)
O5—C9—C5—C4	1.3 (7)	O3^iii^—Na1—O1—C7	−63.3 (13)
C5—C4—C3—C2	0.1 (8)	O3W—Na1—O1—C7	27.6 (13)
C5—C4—C3—C8	−177.5 (5)	Na1^iv^—Na1—O1—C7	−107.5 (13)
O3—C8—C3—C2	−23.6 (8)	O6—C9—O5—Zn1^v^	4.6 (6)
O4—C8—C3—C2	159.3 (6)	C5—C9—O5—Zn1^v^	−173.8 (4)
O3—C8—C3—C4	154.0 (5)	O1—C7—O2—Zn1	2.3 (6)
O4—C8—C3—C4	−23.1 (9)	C1—C7—O2—Zn1	−178.7 (4)
C4—C3—C2—C1	0.8 (8)	O2W—Zn1—O2—C7	59.7 (4)
C8—C3—C2—C1	178.3 (5)	O5^i^—Zn1—O2—C7	179.4 (3)
C5—C6—C1—C2	1.0 (8)	O1W—Zn1—O2—C7	−65.9 (4)
C5—C6—C1—C7	179.3 (5)	O4—C8—O3—Na1^vi^	−82.2 (8)
C3—C2—C1—C6	−1.3 (8)	C3—C8—O3—Na1^vi^	100.7 (5)
C3—C2—C1—C7	−179.5 (5)	O4—C8—O3—Na1^iii^	119.5 (6)
O1—C7—C1—C6	−8.2 (8)	C3—C8—O3—Na1^iii^	−57.5 (6)

Symmetry codes: (i) *x*, *y*−1, *z*; (ii) *x*−1/2, −*y*+3/2, *z*−1/2; (iii) −*x*+1, *y*, −*z*+3/2; (iv) −*x*+1/2, −*y*+3/2, −*z*+1; (v) *x*, *y*+1, *z*; (vi) *x*+1/2, −*y*+3/2, *z*+1/2.

### Hydrogen-bond geometry (Å, °)

**Table:**

*D*—H···*A*	*D*—H	H···*A*	*D*···*A*	*D*—H···*A*
O1W—H1WA···O5^vii^	0.93 (5)	2.35 (8)	3.090 (6)	137 (9)
O2W—H2WA···O2^viii^	0.93 (5)	2.28 (9)	2.998 (6)	133 (9)
O3W—H3WA···O6	0.85 (5)	2.11 (7)	2.747 (7)	132 (7)
O4W—H4WB···O4^ix^	1.00 (5)	1.71 (5)	2.686 (8)	166 (8)
O4W—H4WA···O3W^x^	0.85 (4)	2.37 (7)	2.948 (8)	126 (9)

Symmetry codes: (vii) −*x*+1, *y*−1, −*z*+3/2; (viii) −*x*+1, −*y*+1, −*z*+1; (ix) −*x*+1, −*y*+2, −*z*+1; (x) −*x*+1/2, *y*−1/2, −*z*+1/2.
